# Dexamethasone-dependent modulation of cyclic GMP synthesis in podocytes

**DOI:** 10.1007/s11010-015-2528-6

**Published:** 2015-08-14

**Authors:** Barbara Lewko, Anna Waszkiewicz, Anna Maryn, Magdalena Gołos, Elżbieta Latawiec, Agnieszka Daca, Jacek M. Witkowski, Stefan Angielski, Jan Stępiński

**Affiliations:** Department of Pharmaceutical Pathophysiology, Medical University of Gdansk, Debinki 7, 80-211 Gdansk, Poland; Department of Immunopathology, Medical University of Gdansk, Gdansk, Poland; Department of Pathology and Experimental Rheumatology, Medical University of Gdansk, Gdansk, Poland; Department of Pathophysiology, Medical University of Gdansk, Gdansk, Poland; Laboratory of Cellular and Molecular Nephrology, Mossakowski Medical Research Centre, Polish Academy of Sciences, Warsaw/Gdansk, Poland

**Keywords:** Podocytes, Dexamethasone, cGMP, Angiotensin II, Mechanical stress, Motility

## Abstract

Podocytes may be direct target for glucocorticoid therapy in glomerular proteinuric disease. Permeability of podocytes largely depends on their capacity to migrate which involves the contractile apparatus in their foot processes. In this study, we examined the effect of synthetic glucocorticoid dexamethasone (DEX) on the ability of podocytes to produce cyclic guanosine monophosphate (cGMP) in the presence of vasoactive factors, atrial natriuretic peptide (ANP), nitric oxide (NO), and angiotensin II (Ang II). We investigated also the effects of cGMP and DEX on podocyte motility. Primary rat podocytes and immortalized mouse podocytes were pretreated with 1 µM DEX for 4 or 24 h. Glomerular hypertension was mimicked by subjecting the cells to mechanical stress. Total and subcellular cGMP levels were determined in podocytes incubated with 0.1 µM ANP, 1 µM *S*-nitroso-*N*-acetyl penicillamine (SNAP), and 1 µM Ang II. Cell motility was estimated by a wound-healing assay. The ANP-dependent production of cGMP increased after 4 h exposition to DEX, but was attenuated after 24 h. Adversely, a 24-h pretreatment with DEX augmented the NO-dependent cGMP synthesis. Ang II suppressed the ANP-dependent cGMP production and the effect was enhanced by DEX in mechanical stress conditions. Mechanical stress reduced total cGMP production in the presence of all stimulators, whereas extracellular to total cGMP ratio increased. 8-Br cGMP enhanced podocyte migration which was accompanied by F-actin disassembly. In the presence of DEX these effects were prevented. We conclude that DEX modulates the production of cGMP in podocytes stimulated with vasoactive factors such as Ang II, ANP, and NO, and the effect is time-dependent. cGMP increases podocyte motility, which is prevented by DEX. This mechanism may account for the antiproteinuric effect of glucocorticoids.

## Background

Glucocorticoids are steroid hormones that are widely used to treat both inflammatory and non-inflammatory glomerular diseases. The canonical model of glucocorticoid function is based on glucocorticoid receptor (GR)-mediated changes in gene expression resulting in modulation of immune response. However, now it is clear that cellular response to glucocorticoids is more complex and involves also GR-independent as well as non-genomic GR-dependent mechanisms [1, 2]. Glucocorticoid therapy has remained a mainstay treatment for nephrotic syndrome (NS) for more than 50 years, although precise mechanisms of action of these drugs in NS are not clear. Recent evidence shows that NS, a disorder of glomerular filtration barrier, is associated with impairment of podocytes that appear to be a major direct target for glucocorticoid therapy [3, 4]. Podocytes are highly differentiated cells that attach to the outer surface of glomerular basement membrane (GBM) by foot processes containing actin-based contractile apparatus. This dynamic cytoskeleton allows the cells to migrate along the GBM, which in physiologic conditions is an adapter mechanism stabilizing the glomerular filter. A typical feature of NS is podocyte foot process effacement and disruption of bridging slit diaphragm, with resulting massive proteinuria. Recent findings indicate that effacement of foot processes is strictly associated with changes in focal adhesions and migratory phenotype of podocytes [5]. It has been proposed recently that glucocorticoids directly stabilize actin filaments in podocytes, thus enhancing recovery of these cells in NS [6]. Since dynamic changes in actin structure underlie the cell motility, it seems likely that antiproteinuric effects of glucocorticoids may involve changes in migratory properties of podocytes.

Contractility and structural proteins of foot processes are regulated by mechanical stress and by vasoactive hormone systems, many of which are modulated in response to podocyte strain [7–9]. Among numerous vasoactive substances shown to interact with podocytes, angiotensin II (Ang II) and factors signaling through cGMP pathway seem to play a pivotal role in the functioning of these cells [10–12]. Apart from expressing receptors for respective ligands, podocytes possess their intrinsic renin-angiotensin and natriuretic peptide (NP) systems that may act in these cells in a paracrine as well as in an autocrine manner [8, 13]. Both Ang II and cGMP systems, by counteracting each other, provide a balance between constricting and relaxing forces, which presumably regulates the ultrafiltration coefficient K_f_ [14, 15]. The cGMP system comprises two different guanylyl cyclase (GC) enzyme families, i.e., nitric oxide (NO)-dependent soluble GC (sGC) and NP-dependent particulate GC (pGC). We have reported previously that injections of DEX sensitized the sGC-dependent cGMP production in rat kidney glomeruli, while pGC response to atrial natriuretic peptide (ANP) was blunted [16, 17].

In the present study, we show that glucocorticoids directly regulate the response of glomerular podocytes to vasoactive factors and that the effect varies dependent on the time of exposition to the steroid. We examined the effects of DEX on the ability of cultured rat and mouse podocytes to generate cGMP in the presence of pGC and sGC agonists and we examined if DEX influenced the interaction between cGMP- and Ang II-dependent systems. In order to assess the physiologic consequences of the interaction between glucocorticoids and cGMP, we investigated the effects of both these compounds on podocyte motility.

## Materials and methods

### Isolation of glomeruli and primary culture of rat podocytes (RP)

All procedures that involved animals were performed according to EU guidelines and approved by local Bioethical Commission at University of Gdansk.

Wistar rats, weighing 120–160 g, with free access to the standard diet (Altromin C1324, Germany) and drinking tap water were anesthetized as described before [18]. Glomeruli isolated from renal cortex by gradual sieving were incubated for 5 days at 37 °C in RPMI1640 (Sigma-Aldrich, Poland) containing 10 % heat-inactivated fetal bovine serum (FBS, Pan-Biotech, Germany), 100 U/ml penicillin, and 0.1 mg/ml streptomycin (Sigma-Aldrich, Poland). The outgrowing epithelial cells were trypsinized, passed through a sieve with 33 μm pore size, seeded in 12-well or 6-well plates, and cultivated at 37 °C for next 15–20 days, as described previously [17]. The seeding density was 50 × 10^3^ cells/cm^2^. Cell identity and phenotype were confirmed by immunofluorescence, using antibodies to WT-1 (Santa Cruz Biotechnology), podocin (Sigma-Aldrich, Poland), and synaptopodin (Progen, Germany).

### Culture of immortalized mouse podocytes (MP)

Conditionally immortalized mouse podocytes (Clone SVI, generous gift from Dr N. Endlich, Greifswald University, Germany) were cultured as described before [19]. To propagate, the cells were cultivated at 33 °C in a humid atmosphere with 5 % CO_2_, in RPMI1640 supplemented with 10 % FBS, 100 U/ml penicillin, 0.1 mg/ml streptomycin, and 10 U/ml mouse recombinant γ-interferon (Sigma-Aldrich, Poland). Differentiation was induced by shifting the cells to 37 °C and the culture was continued for next 10–14 days in the absence of γ-interferon, with FBS concentration reduced to 5 %.

## Experimental procedures

Before stimulation of cGMP production, the podocytes grown in 12- or 6-well plates were preincubated with 1 μM dexamethasone (DEX, Dexaven, Jelfa-Poland) at 37 °C for 4 or 24 h, with or without 10 μM RU 486 (Sigma-Aldrich, Poland, Poland), a specific antagonist of glucocorticoid receptors. Final DEX concentration was selected based on previously published reports [4, 20]. To avoid the potential influence of serum-derived steroids, preincubations were conducted in RPMI1640 containing antibiotics and 2 % bovine serum albumin (BSA, Sigma-Aldrich, Poland) instead of FBS. One hour prior to stimulation, the medium was supplemented with 1 mM 3-isobutyl-1-methylxanthine, a phosphodiesterase inhibitor (IBMX, Sigma-Aldrich, Poland). Stimulation of sGC was started by adding a nitric oxide donor, 1 μM *S*-nitroso-*N*-acetyl penicillamine (SNAP), while pGC was stimulated with 0.1 μM ANP (both activators from Sigma-Aldrich, Poland). In some experiments, 1 μM C-ANP_4-23_ (Bachem, Germany), a specific ligand for natriuretic peptide clearance receptor (NPR-C) was added one minute prior to ANP. Cells incubated with respective volumes of DMSO served as vehicle controls. 1 μM Ang II (Sigma-Aldrich, Poland) was added simultaneously with ANP. Basal cyclic GMP level was determined in the wells to which corresponding volume of incubation medium without activators was added. The 15-min incubations at 37 °C were terminated with addition of 6 % (f.c.) ice-cold perchloric acid. The supernatants were transferred to eppendorf tubes, neutralized, frozen, and stored at −20 °C for total cGMP determination. Remaining cells were solubilized in 350 µl 0.01 % SDS in 0.01 N NaOH and 50 μl aliquots were used for protein assay. In experiments in which distribution of cGMP was studied, incubations were terminated by placing the culture plates on ice. The supernatants were immediately transferred to the eppendorf tubes for extracellular cGMP assay while remaining cells were rinsed twice with an ice-cold PBS containing 100 μM IBMX and 100 μM probenecid (Sigma-Aldrich, Poland.) to prevent further cGMP extrusion. Subsequently, 300 μl H_2_O with 1 mM IBMX was added to each well for 30 min to extract the intracellular cGMP.

### Mechanical stress experiments

Differentiated rat podocytes were grown in six-well culture plates with a flexible bottom coated with collagen I (Bioflex, Flexcell International, Hillsborough, NC, USA). 60 min prior to the mechanical stress, culture media were replaced by RPMI1640 containing 2 % BSA instead of FBS, with or without 1 μM DEX. The plates were mounted on a manifold connected to the stretch apparatus (StretchCo, Edingen, Germany). Variations in air pressure caused cyclic oscillations of the flexible plate bottom, with frequency adjusted to 0.5 Hz and maximum linear strain 5 % [21]. The cells were subjected to mechanical strain for 24 h, while podocytes grown in wells with flexible membranes not subjected to mechanical stress served as controls.

### Measurements of cGMP and protein content

 cGMP was determined in duplicate by the modified radioimmunoassay method [22], using rabbit anti-cGMP antibodies and 3′,5′ cyclic (8,5′-3H) guanosine monophosphate ammonium salt, (DuPont NEN Products Boston, MA, USA) as a radioligand [23]. Radioactivity was detected in the liquid scintillation counter (Wallac 1409). Protein content was determined in triplicate according to the modified Bradford method [24], using bovine serum albumin as a standard.

### Quantitative real-time PCR

Differentiated mouse podocytes were incubated for 4 or 24 h with 1 μM DEX, as described above. Total RNA was extracted using TRI Reagent (Sigma-Aldrich) according to the manufacturer’s instructions. One microgram of total RNA was reverse transcribed using 0.5 μg of random hexamer primers (EURx, Poland), 0.5 mM dNTP mixture (Sigma-Aldrich, Poland), 10 U/µl MuLV reverse transcriptase (Promega, Madison, WI, USA), and 1 U/µl ribonuclease inhibitor. Primer sequences are shown in Table 1. The amplification conditions were as follows: activation at 95 °C for 2 min followed by 50 cycles of denaturation at 95 °C for 10 s, annealing at 58 °C for 20 s, and extension at 72 °C for 30 s. Under optimized conditions there was a single melting curve and no primer-dimer formation. The housekeeping gene GAPDH was amplified to confirm there was no change in expression level under our experimental conditions. Experiments were performed in triplicate for each gene and were repeated three times using independent biological replicates. Thermo Scientific PikoReal 96 cycler was used to analyze the gene expression level, and PikoReal Software 2.1 (ThermoScientific) was used to analyze the results. All of the results are expressed as ratio to GAPDH.

**Table 1:** Mouse primer sequences used for real-time PCR

Gene	Sequence of primers (5′–3′)	Accession #
NPR-A	SE: CTCAACATCACAGTAAATCACC AS: CCTGAAGGCACCTGTCTCG	NM 008727.5
NPR-C	SE: CTTCCAGGTGGCCTACGAA AS: GGCACACATGATCACCACTC	NM 008728.2
GAPDH	SE: TGAAGGTCGGTGTGAACGGATTTG AS: ACATTGGGGGTAGGAACACGGAAGG	NM 008084.2

Previously published primer sequences were verified by NCBI BLAST. Amplification was conducted for 50 cycles

*SE* sense, *AS* antisense

### Flow cytometry

The cells were washed with PBS, trypsinized, suspended in PBS, and centrifuged two times for 7 min at 400×*g* at room temperature. This was followed by incubation in blocking solution (2 % FBS, 2 % bovine serum albumin, 0.2 % fish gelatine, in PBS) for 60 min. After washing with PBS, the cells were incubated in room temperature for 60 min with primary rabbit antibodies to NPR-A and NPR-C (1:100, Santa Cruz Biotechnology, Inc.). Antigen-bound antibodies were visualized by 45 min incubation at +4 °C with Alexa Fluor488-conjugated donkey anti-rabbit IgG (1:100, Sigma-Aldrich, Poland). Stained cells were washed with 1 ml PBS, resuspended in 300 μl PBS containing 10 % FBS, and analyzed in flow cytometer (FACScan Flow Cytometer, BD, USA).

### Scrape-wound assay

Differentiated sub-confluent mouse podocytes were grown on glass coverslips placed in the 24-well plates. Prior to the incubations, the cells were serum-starved, as mentioned before. Experiment was started by scratching the coverslips with a 20 µl sterile pipette tip. After replenishing the wells with fresh media, 4- or 24-h incubations with 1 µM DEX and/or 100 μM 8-Br cGMP (Sigma-Aldrich, Poland) were performed. Subsequently, the cells were fixed with 2 % paraformaldehyde, permeabilized with 0.3 % Triton X-100 in PBS, and stained with DAPI (Sigma-Aldrich, Poland) and Phalloidin-Alexa488 conjugate (Molecular Probes-Life Technologies, Poland). At 0 and 24 h after scratching, pictures were taken under fluorescent microscope (Olympus IX51), using CellSens v.1.3 imaging software (Olympus). In each coverslip, the number of cells that have migrated into same-sized fields was counted in 3 different points. The data presented represent the mean from 3 independent experiments performed in duplicate.

### Statistics

Unless stated otherwise, cGMP levels are shown as a difference between drug-stimulated and basal values. Data are presented as a mean value ±SEM. Statistical analysis was performed using SigmaPlot version 11.0 software (SPSS Inc. USA). The differences between two groups were analyzed by *t* test, and multiple groups were compared using two-way ANOVA. *P* < 0.05 was considered statistically significant.

## Results

### Dexamethasone differentially regulates ability of podocytes to produce cGMP in a time-dependent manner

**Fig. 1:**
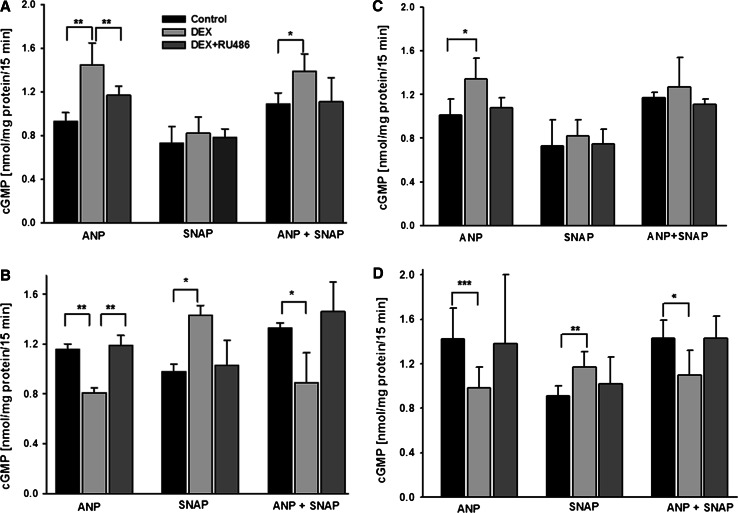
Ability of mouse (**a** and **b**) and rat (**c** and **d**) podocytes to produce cGMP varied depending on the time of exposure to 1 µM DEX. The cells cultured in 12-well plates were stimulated with 0.1 µM ANP or 1 µM SNAP for 15 min. **a** and **c** cGMP synthesis increased in ANP-stimulated cells after a 4-h preincubation with DEX. **b** and **d** Following a 24-h preincubation with DEX, the ANP- dependent cGMP production was suppressed, while SNAP-induced cGMP was elevated, as compared to the controls. Co-incubation of podocytes with ANP and SNAP did not reverse the inhibitory effect of DEX on ANP-induced synthesis of cGMP. The DEX-dependent effects were abolished in the presence of RU486, a specific GR antagonist. Results are presented as means from 5 to 6 independent experiments performed in duplicate ±SEM. *P < 0.05, **P < 0.01, ***P < 0.001

A 24-h exposure of mouse podocytes to 1 μM DEX reduced their basal (non-stimulated) cGMP synthesis by almost 50 % (0.61 ± 0.02 vs. 1.19 ± 0.01 nmol/mg protein, *P* < 0.01). Similar effect was observed in rat cells. DEX affected also the ability of podocytes to produce cGMP in the presence of ANP and SNAP, albeit the effects varied depending on preincubation time (Fig. 1). The ANP-mediated cGMP formation was markedly enhanced by DEX after a short-time (4-h) preincubation (Fig. 1a, c), whereas adverse effect was observed after prolonged (24-h) DEX pretreatment (Fig. 1b, d). Moreover, in both mouse and rat cells, DEX-induced suppression of response to pGC activator ANP was accompanied by increased response to sGC activator, SNAP. Interestingly, when podocytes were incubated with both activators simultaneously, cGMP production remained suppressed in the cells pretreated for 24 h with DEX. This indicates that upregulation of sGC-derived cGMP did not compensate for decreased production of cGMP by pGC (Fig. 1b, d). Pretreatment of podocytes with RU486 abolished the effects of DEX in both, rat and mouse podocytes (Fig. 1). This suggests that modulation of cGMP level in these cells was mediated by glucocorticoid receptors (GR).

### Effect of dexamethasone on natriuretic peptide receptors (NPRs)

**Fig. 2:**
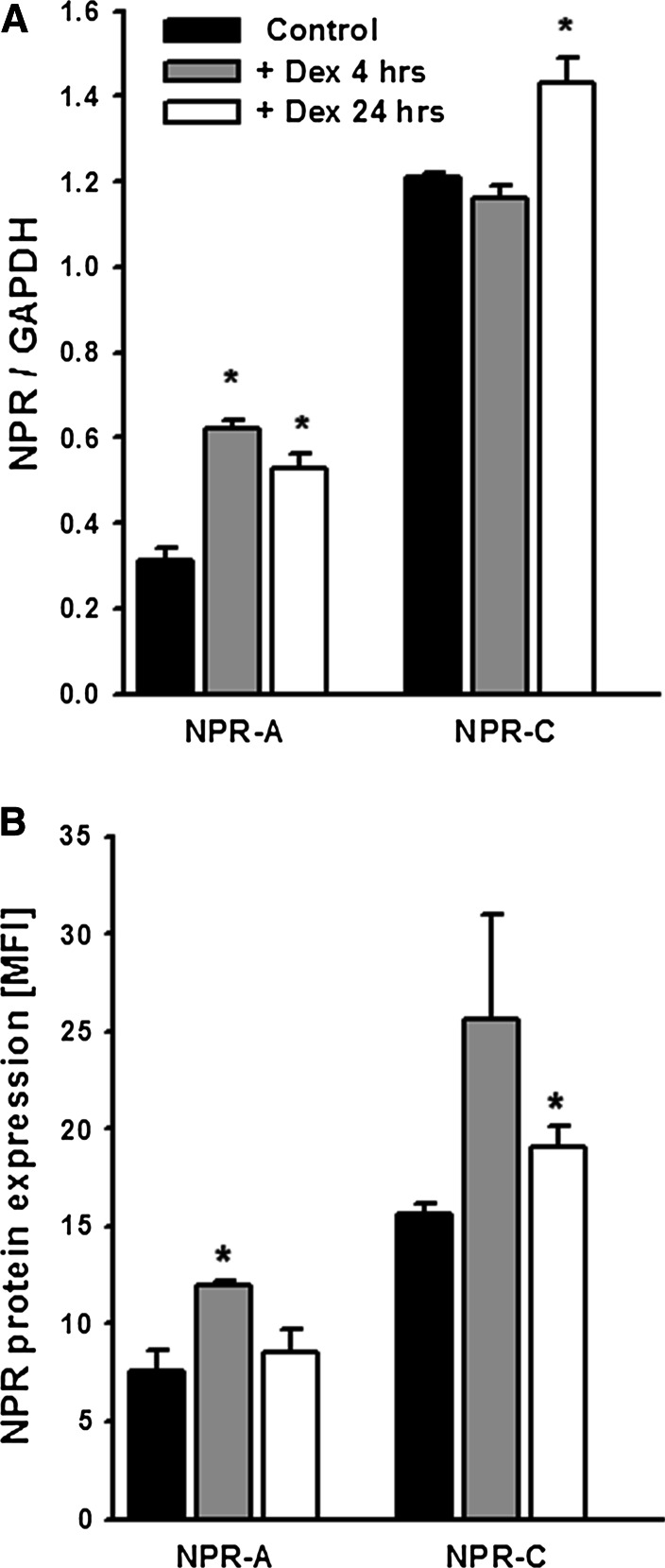
**a** Preincubation of mouse podocytes with 1 µM DEX upregulated the NPR-A mRNA already after 4 h, whereas increase in NPR-C mRNA could be observed after 24 h. Quantitative real-time PCR of NPR levels were normalized to glyceraldehyde 3-phosphate dehydrogenase (GAPDH). **b** Similarly to mRNA, expression of NPR-A and NPR-C protein increased upon DEX treatment. Flow cytometry results are expressed as the mean fluorescence intensity (MFI). All results are presented as means from three independent experiments ± SEM. *P < 0.05 versus control

Since the canonical mechanism of action of glucocorticoids is based on their genomic actions, we have tested if observed by us effects of DEX on cGMP response to ANP could result from respective changes in the expression of NP receptors. As shown in Fig. 2a, quantitative real-time PCR analysis revealed that mRNA for NPR-C in mouse podocytes significantly raised after 24 h treatment with DEX (1.43 ± 0.06 vs. 1.21 ± 0.01, *P* < 0.05 vs. control) while there was no change after 4 h. Similarly to mRNA, marked NPR-C protein elevation (19.1 ± 1.0 vs. 15.6 ± 0.6, *P* < 0.05 vs. control) could be observed after 24-h but not after 4 h incubation with DEX (Fig. 2b). The mRNA levels for NPR-A, a pGC-linked receptor doubled after 4 h incubation with DEX (0.62 ± 0.02 vs. 0.31 ± 0.03, *P* < 0.05 vs. control) which was accompanied by corresponding increase in NPR-A protein expression (12 ± 0.2 vs. 7.6 ± 1.1, *P* < 0.05 vs. control). Elevated NPR-A expression after 4 h as well as increased NPR-C expression after 24 h correlated with observed by us respective changes in the ANP-dependent cGMP production at the same time (Fig. 1).

**Fig. 3:**
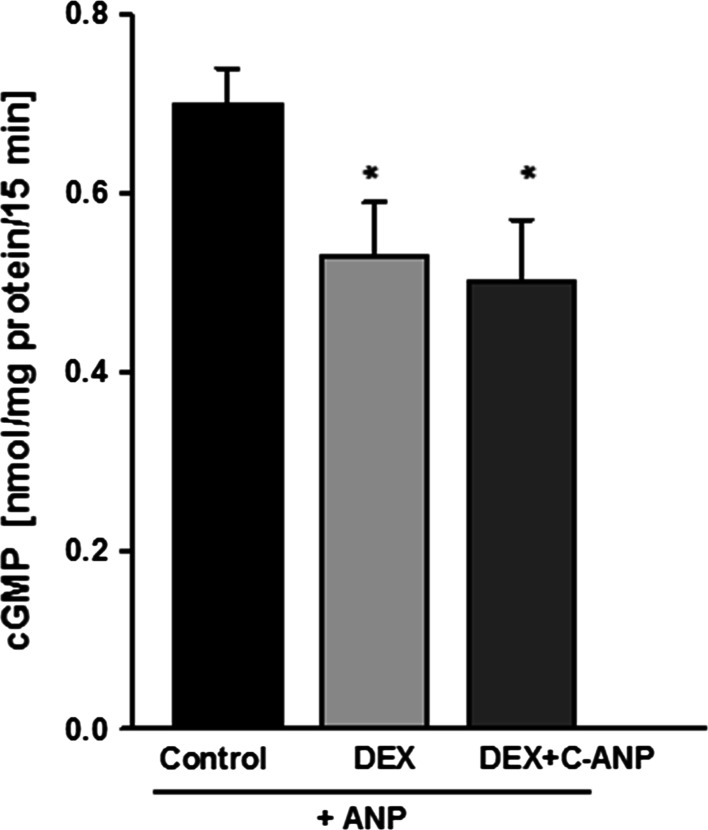
Blocking of NPR-C receptors with C-ANP_4-23_ did not influence the inhibitory effect of DEX on ANP-stimulated cGMP synthesis in mouse podocytes. The cells were preincubated for 24 h with DEX, and C-ANP_4-23_ was added 1 min prior to the 15-min stimulation with ANP, as indicated in Materials and Methods. Results are presented as means from four independent experiments ±SEM. *P < 0.05 versus control

Posttranscriptional modulation of NPR-C could be another possible mechanism by which DEX could influence the activity of ANP in podocytes. To test this possibility, prior to incubation with ANP the cells were pretreated with C-ANP_4-23_, a ring-deleted analog of ANP that specifically interacts with clearance natriuretic peptide receptor [25]. However, as shown in Fig. 3, blocking of NPR-C receptor had no effect on the inhibitory action of DEX on the ANP-dependent production of cGMP.

### Dexamethasone potentiates the inhibitory effect of Ang II on ANP-dependent synthesis of cGMP in mechanically stressed podocytes

We have previously shown that in cultured rat and mouse podocytes stimulated with ANP, Ang II via its AT1 receptors decreased cGMP synthesis [14, 17]. On the other hand, upregulation [26, 27] or downregulation [28, 29] of AT1 receptors by DEX has been reported in different cell types. Therefore, we have investigated if a 24-h exposition of podocytes to DEX modulates the effect of Ang II on the response of the cells to ANP.

**Fig. 4:**
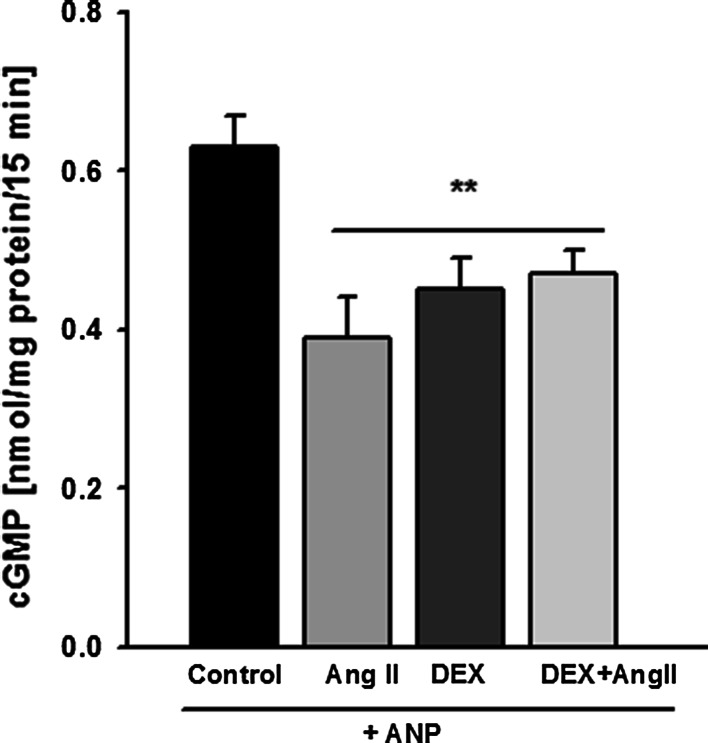
Dexamethasone did not influence the inhibitory effect of Ang II on ANP-dependent production of cGMP. Mouse podocytes were preincubated for 24 h in the presence or absence of 1 µM DEX. 0.1 μM ANP and/or 1 μM Ang II were added for 15 min, as described in “Materials and methods.” Results are presented as means from 4 independent experiments ±SEM. **P < 0.01 versus control

Figure 4 demonstrates that incubation with Ang II of the DEX-pretreated mouse podocytes did not change significantly the ANP-induced cGMP production, as compared to the cells exposed to Ang II or to DEX alone. While both, Ang II and DEX separately decreased the cGMP level, their effects were not additive. Similar results were obtained in rat podocytes (Table 2, non-stressed NS cells). However, elevated intrarenal Ang II levels in vivo are frequently associated with glomerular hypertension, which exposes podocytes to additional mechanical forces. In order to mimick these conditions, we applied mechanical strain and we examined whether it affected the cGMP response in the presence of Ang II and DEX. Cultured rat podocytes were subjected for 24 h to mechanical stress with or without DEX, and the effect of Ang II on ANP-dependent cGMP production was measured. As shown in Table 2, similarly to NS podocytes, Ang II markedly inhibited the effect of ANP in the stressed (S) cells. Moreover, in contrast to NS group, DEX further significantly suppressed the cGMP level in the presence of Ang II. These findings suggest that mechanical stress and DEX synergistically sensitized the podocytes to Ang II.

**Table 2:** Effect of dexamethasone (DEX) and mechanical stress on cGMP distribution in cultured rat podocytes

		NS—non-stressed cells		S—stressed cells	
		Control	+DEX	Control	+DEX
		nmol cGMP/10^5^ cells/15 min			
ANP	[cGMP]_t_	89 ± 10	44 ± 5^#^	70 ± 7*	51 ± 6^#^
	[cGMP]_e_	16 ± 3	14 ± 3	39 ± 6^*^	30 ± 1
	[cGMP]_e_ (%) [cGMP]_t_	18 ± 3	38 ± 5^#^	52 ± 4^*^	61 ± 6^*^
ANP+Ang II	[cGMP]_t_	66 ± 3^†^	57 ± 3	49 ± 7^†^	26 ± 5^#*$^
	[cGMP]_e_	14 ± 2	19 ± 2	34 ± 3^*^	15 ± 2^#^
	[cGMP]_e_ (%) [cGMP]_t_	21 ± 3	28 ± 4	72 ± 5^†*^	56 ± 2
SNAP	[cGMP]_t_	20 ± 3	31 ± 2^#^	11 ± 2*	9 ± 2^*^
	[cGMP]_e_	8 ± 1	14 ± 7	9 ± 2	7 ± 1^*^
	[cGMP]_e_ (%) [cGMP]_t_	34 ± 2	36 ± 7	78 ± 5^*^	81 ± 4^*$^

The podocytes cultured in 6-well plates with flexible bottom were mechanically stressed for 24 h with or without (control) 1 μM DEX. ANP, SNAP, and Ang II were added for 15 min as described in Materials and Methods. [cGMP]_e_—extracellular cGMP, [cGMP]_t_—total cGMP. Results are presented as means from 3 to 4 independent experiments ±SEM

* *P* < 0.05 versus respective non-stretched cells

^#^ * P* < 0.05 versus respective control cells

^†^ * P* < 0.05 versus respective cells without Ang II

^$^ * P* < 0.05 versus respective (ANP + DEX) cells

### Effect of dexamethasone and mechanical stress on distribution of cGMP in podocytes

Feedback mechanisms associated with changes of intracellular cGMP level could account for regulation of ANP- and SNAP-mediated total cGMP production. Therefore, we tested if observed by us effects were accompanied by respective changes of cGMP compartmentation in podocytes pretreated for 24 h with DEX. Furthermore, we tested if mechanical stress, a potent factor inducing transcriptional changes in podocytes [30], could influence the effects of DEX on cGMP production.

As shown in Table 2, in the SNAP-treated group, DEX did not affect the [cGMP]_e_ to [cGMP]_t_ ratio which suggests that proportionally, intracellular cGMP level remained unchanged. In contrast, in ANP-treated podocytes from NS group the ratio was elevated by DEX, which indicates that cGMP transport outward the cells was increased. This, however, is in contradiction to the hypothesis that high intracellular cGMP might be responsible for drop in cGMP production in these cells. Mechanical stress decreased total cGMP production in all control groups. Pretreatment with DEX decreased the ANP-dependent production of cGMP in both, NS and S podocytes. Yet, in contrast to the NS cells, DEX failed to elevate the SNAP-induced cGMP in stretched podocytes. Remarkably, compared with respective NS cells, there was a significant increase in the extracellular to total cGMP ratio in all stressed groups. This indicates that mechanical stress alone induces extrusion of cGMP from podocytes. Yet, in the presence of Ang II, addition of DEX suppressed both total and extracellular cGMP in podocytes stimulated with ANP.

### Dexamethasone prevents the cGMP-induced motility of mouse podocytes

**Fig. 5:**
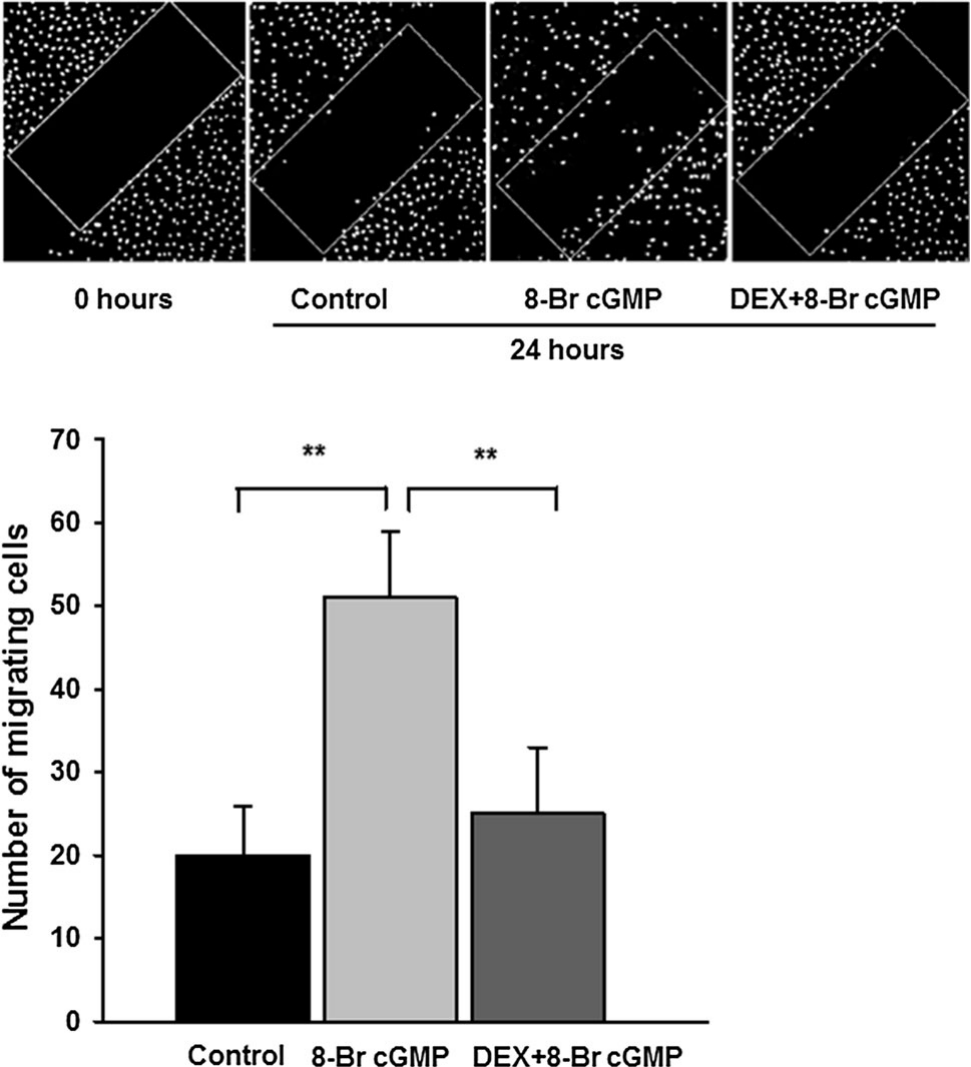
The influence of 8-Br cGMP and DEX on mouse podocyte motility. *Upper panel* scrape-wound assay, DAPI staining. The serum-starved podocytes were incubated for 24 h with 100 µM 8-Br cGMP with or without 1 µM DEX, as described in “Materials and methods.” Untreated cells served as the control. *Lower panel* the results showing that 8-Br cGMP significantly increased the number of migrating podocytes, while DEX inhibited this effect. The data represent the mean ±SEM from three independent experiments performed in duplicate

**Fig. 6:**
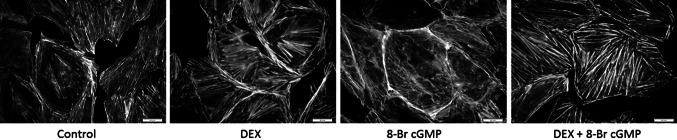
Effect of 8-Br cGMP and DEX on the F-actin architecture in mouse podocytes. The 24-h incubation with 8-Br cGMP resulted in a pronounced disassembly of the transcellular stress bundles, accompanied by the concentration of fibers in cortical regions. In the presence of DEX with or without 8-Br cGMP, F-actin structure remained unaffected and was similar to the structure in the control cells

Since the involvement of guanylyl cyclases and glucocorticoids in migration of some cell types has been reported previously [31, 32], we have tested if motility of podocytes is regulated by these factors. Due to phosphodiesterases activity, half-time for cGMP varies between few seconds in vivo to few minutes in vitro. Therefore, in order to perform prolonged incubations, we used a membrane-permeable analog 8-Br cGMP. While no significant effects were observed after a 4-h incubation with 8-Br cGMP, a 24-h incubation resulted in markedly higher (*P* < 0.01) ability of podocytes to migrate, as compared to the non-treated (control) cells (Fig. 5). This was accompanied by a pronounced disassembly of F-actin fibers (Fig. 6). As shown in Fig. 5, incubation with DEX prevented the cGMP-dependent increase in podocyte motility, while F-actin stress fibers remained well preserved, forming a strong network (Fig. 6).

## Discussion

Recent reports have emphasized the role of podocytes as a key cell type in proteinuria, a condition which is particularly profound in NS [3]. Moreover, a number of the in vivo and in vitro studies have shown that the effectiveness of glucocorticoid therapy in NS may be related to their effects on podocyte structure and functions [33–35].

In the present study, we demonstrated that in rat and mouse podocytes, synthetic glucocorticoid DEX strongly modulated the cGMP response to ANP, a pGC activator and to SNAP, a donor of nitric oxide that activates sGC. The effects varied depending not only on the time of exposition of the cells to DEX but also on isoform of activated GC. After a short-term (4-h) preincubation with DEX, the cGMP response of podocytes to ANP increased by 36 % in rat and by 56 % in mouse cells, while the response to SNAP remained unaffected. Conversely, a prolonged (24-h) exposition of the rat and murine podocytes to DEX decreased the ANP-dependent cGMP production by 30 %, whereas increase of cGMP was observed in SNAP-treated cells (Fig. 1). We previously showed that in the glomeruli of rats receiving DEX, sensitivity of sGC to nitric oxide was elevated, which was accompanied by the decrease of pGC response to ANP [16]. Our present in vitro study has let us to follow more precisely direct effects of DEX on glomerular podocytes. It appeared that direction of changes in cGMP production depends on the time of exposition to the glucocorticoid. The mechanisms underlying such differential regulation of pGC and sGC -dependent cGMP production are not clear. It is possible that there is a strict compartmentation of the pGC and sGC systems in podocytes and that DEX could enhance cGMP production by of one of these systems. As it was shown in rat cardiomyocytes [36], activation by cGMP protein kinase (PKG) could exert a feedback control of total cGMP resulting in the diverse accumulation of cGMP induced by ANP and by SNAP.

All DEX-dependent changes were abolished in the presence of a glucocorticoid receptor antagonist RU486 (Fig. 1) which indicates that they were mediated by glucocorticoid receptor. Some previous studies have already confirmed the presence of glucocorticoid receptors in podocytes [4, 37, 38] that may act through genomic, as well as through non-genomic pathways. In our experiments, varying with the time of exposure to DEX changes in NPR-A and NPR-C expression (Fig. 2) correlated with the changes in ANP-stimulated production of cGMP (Fig. 1). This suggests that glucocorticoid regulated the cGMP response to ANP via modulating the NPR availability. The time dependency of mouse podocyte response to DEX has been reported recently [20]. This was associated with transient GR phosphorylation which could modulate the receptor activity [39]. Similar mechanism could be responsible for observed by us time-dependent effects of DEX on NPR and cGMP levels in podocytes. Upregulation by glucocorticoids of NPR-A and NPR-C protein in various tissues including renal cells was reported previously [43, 44]. However, the consequences of the changes in cGMP production were diverse and seemed to depend on the cell type [45, 46]. Observed in our experiments varying levels of cGMP could also reflect the DEX-induced changes in the activities of the systems involved in its production and/or degradation. Lack of effects of NPR-C blocker C-ANP_4-23_ (Fig. 3) suggests that attenuated cGMP responses to ANP could depend not only on the activity of clearance receptor but could also reflect modulated by DEX activity of phosphodiesterases. However, this concept seems to be less likely because all experiments were conducted in the presence of a widely used phosphodiesterase inhibitor IBMX [17, 36]. Moreover, after 24-h treatment with DEX, the ANP-dependent cGMP was decreased but simultaneously, SNAP-dependent cGMP was markedly elevated (Fig. 1b, d). Increased cGMP response to SNAP was apparent after a 24-h exposure to DEX which suggests that the effect could be mediated by changes in protein expression. It is possible that similarly to other glomerular cells [40], glucocorticoid induced superoxide dismutase (SOD) in podocytes. As a potent antioxidant, SOD could protect intracellular NO from being scavenged by superoxide and thus, stimulation of sGC could be augmented. Decreased cGMP response to ANP could also result from upregulation by DEX of neutral endopeptidase (NEP), an enzyme that degrades natriuretic peptides [41] and is expressed on podocyte surface [13, 42].

One of the canonical functions of cyclic GMP is to mediate relaxatory effects of vasoactive factors. In physiologic conditions, these effects are balanced by vasoconstricting hormones, of which angiotensin II (Ang II) plays a pivotal role. Glucocorticoids have been shown to regulate the effects of Ang II, although the role of these hormones seems to depend on the cell type [26, 29]. In our previous [14] as well as in the present experiments with the cells cultured in stationary conditions, Ang II decreased the ability of podocytes to generate cGMP in response to ANP. As shown in Fig. 4, pretreatment of the cells with DEX did not significantly influence this effect. However, in podocytes subjected to mechanical stress, pretreatment with DEX markedly enhanced the inhibitory action of Ang II (Table 2). Exposition of podocytes to increased Ang II concentrations in vivo usually occurs in pathologic conditions such as glomerular capillary hypertension. Resulting distension of capillaries causes mechanical strain on foot processes adhering to their surface, which additionally upregulates podocytic local renin-angiotensin system, including expression of AT1 receptors [9]. It was, therefore, reasonable to expect that in our experiments, the response to Ang II in stretched podocytes will be more pronounced than in the NS cells. Yet, observed by us levels of inhibition by Ang II of ANP-induced cGMP were similar (by approximately 30 %) in both NS and S groups. It should be noted that, similarly to our previous observations, mechanical stress diminished the cGMP response to ANP [8], while Ang II further exaggerated this effect.

It is noteworthy that in contrast to the NS group, in mechanically stressed podocytes majority of cGMP was extruded to the extracellular space (Table 2). Cyclic GMP can be actively transported from the intracellular compartment to extracellular space through membrane transport proteins, including probenecid-sensitive organic transporter1 (OAT1) [47]. Increase in OAT1 expression has been demonstrated in vivo in rats receiving DEX [48]. Our results show that in cultured rat podocytes, only in ANP-treated NS cells the extracellular to total cGMP ratio was increased by DEX. Therefore, it seems likely that it was DEX-dependent drop in total cGMP level rather than OAT1 upregulation which was responsible for increased [cGMP]_e_ to [cGMP]_t_ ratio in these cells. On the other hand, as mechanical stress regulates a number of genes in podocytes [7, 30], upregulation of OAT1 in S cells could be responsible for the high percentage of extracellular cGMP in this group.

Mechanisms mediating the beneficial effects of glucocorticoids in podocyte-related proteinuric disease are still a subject of extensive investigations. Traditionally accepted immunosuppressive and antiinflammatory properties of these hormones do not explain their effects in non-inflammatory diseases. We have demonstrated that cGMP stimulated migration of podocytes while DEX counteracted this effect. As shown in Fig. 6, staining of F-actin cytoskeleton revealed that DEX prevented a pronounced disassembly of transcellular stress fibers in cGMP-treated podocytes. Similar microfilament dissociation and cytoskeletal reorganization triggered by cGMP-dependent protein kinase PKG1 was associated with migration of smooth muscle cells [49]. Moreover, decrease of actin stress fibers in podocyte foot processes switches them into a migratory phenotype, which is associated with pedicle effacement, slit diaphragm disruption, and proteinuria [50]. Hence, observed by us effects of DEX on cGMP production, modulation of the cGMP-mediated reorganization of actin filaments and motility may comprise the mechanisms underlying antiproteinuric effects of glucocorticoid therapy. In mechanically stressed podocytes, reorganization of actin cytoskeleton and formation of stress fibers represents an adaptive mechanism, most likely protecting the cells from damage in hypertensive glomeruli [51]. Ang II, via AT1 receptors, may not only increase but also decrease the podocyte contractility, motility, and permeability to proteins which is mediated by a dual antagonistic pathway [52]. It seems plausible that this regulation comprises also inhibition by Ang II of cGMP signaling. Augmentation by DEX of the suppressing effect of Ang II on cGMP formation may favor the antimigratory phenotype of mechanically stressed podocytes. However, since podocyte foot process effacement may result from too much as well as from too little motility [53], it is not clear if the DEX-induced effect is beneficial in hypertension, when activity of Ang II system is increased.

In summary, the present study demonstrates that in cultured rat and mouse podocytes DEX displays time-dependent adverse effects on stimulated formation of cGMP. The effect on ANP-dependent cGMP synthesis involves modulation by DEX of NP receptors. In conditions mimicking glomerular hypertension, DEX augments the inhibitory effect of Ang II on cGMP production. Apart from modulation of podocyte response to vasoactive factors, DEX counteracts the cGMP-induced podocyte migration and F-actin disassembly, which may affect permeability of these cells to proteins. Molecular mechanisms underlying these effects need to be examined in further studies.
